# The Contribution of LATE‐NC to Neuropsychiatric Symptom Burden in Alzheimer's Disease and Lewy Body Disease: A Clinicopathological Study

**DOI:** 10.1002/alz70857_105745

**Published:** 2025-12-26

**Authors:** Yara Alkhodair, Imogene M Scott, Abdulrahman Alzahrani, Paula Villela Nunes, Ian R MacKenzie, Ging‐Yuek Robin Hsiung

**Affiliations:** ^1^ Division of Neurology, Department of Medicine, University of British Columbia, Vancouver, BC, Canada; ^2^ Djavad Mowafaghian Centre of Brain Health, Vancouver, BC, Canada; ^3^ Vancouver Coastal Health, Vancouver, BC, Canada; ^4^ Department of Pathology, University of British Columbia, Vancouver, BC, Canada

## Abstract

**Background:**

Limbic‐predominant age‐related TDP‐43 encephalopathy neuropathological change (LATE‐NC) frequently coexists with Alzheimer's disease (AD), Lewy body disease (LBD), and vascular pathology. While neuropsychiatric symptoms in AD/LATE‐NC are beginning to be explored, the impact of LATE‐NC in mixed AD/LBD and LBD is still underexamined. This study investigated neuropsychiatric symptom profiles in individuals with AD, LBD, and mixed AD/LBD with and without LATE‐NC.

**Method:**

In this retrospective cohort study, individuals from the UBC Hospital Clinic for Alzheimer's and Related Disorders (UBCH‐CARD) autopsy database with AD, LBD or both plus LATE‐NC pathology (LATE+, *n* = 42) were matched by age and neuropathology to controls without LATE‐NC (LATE‐, *n* = 38). Neuropsychiatric symptoms were assessed using the Neuropsychiatric Inventory Questionnaire (NPI‐Q) at initial, intermediate, and final visits before death. Outcomes included symptom severity scores at the final visit, odds ratios of symptoms at the final visit and trajectories of symptom progression. Parametric and non‐parametric tests were used to compare symptom severity scores, while linear mixed‐effects models analyzed longitudinal symptom data, adjusting for age, sex, education, and Clinical Dementia Rating Scale (CDR).

**Result:**

Mean age at death was 80.5 years (LATE+) and 77.9 years (LATE‐), and participants were followed on average 5.8 years. Both groups had a high burden of moderate‐to‐severe AD pathology (LATE+ 97.6% vs. LATE‐ 94.4%) and moderate numbers had neocortical LBD (31.7% vs. 41.7%, *p* = 0.50). At the final visit, total NPI‐Q severity scores were similar between groups (5.65 vs. 6.12, *p* = 0.71). Symptom severity scores for apathy (0.73 vs. 1.29, *p* = 0.01), hallucinations (0.24 vs. 0.82, *p* = 0.01), and appetite changes (0.08 vs. 0.41, *p* = 0.04) were lower in LATE+. Odds ratios for symptom presence at the final visit, adjusted for coexisting pathology, remained significant for hallucinations only (0.013, 95% CI 0.001‐0.218, *p* = 0.002). Longitudinal modeling showed no significant differences in total burden of neuropsychiatric symptoms.

**Conclusion:**

LATE‐NC did not appear to increase overall neuropsychiatric symptom burden in this autopsy cohort study of individuals with AD and/or LBD. Hallucinations were less frequent in the LATE+ group, but this may be due to incomplete adjustment for coexisting Lewy Body pathology. Further modelling of longitudinal symptom progression will be presented at the conference.

